# Amphetamine-induced psychosis - a separate diagnostic entity or primary psychosis triggered in the vulnerable?

**DOI:** 10.1186/1471-244X-12-221

**Published:** 2012-12-05

**Authors:** Jørgen G Bramness, Øystein Hoel Gundersen, Joar Guterstam, Eline Borger Rognli, Maija Konstenius, Else-Marie Løberg, Sigrid Medhus, Lars Tanum, Johan Franck

**Affiliations:** 1 Norwegian Centre for Addiction Research (SERAF), Institute of Clinical Medicine, University of Oslo, Oslo, Norway; 2 Department of Clinical Neuroscience, Karolinska Institutet, Stockholm, Sweden; 3 Department of Biological and Medical Psychology, University of Bergen, Bergen, Norway

**Keywords:** Drug induced psychosis, Amphetamine, Methamphetamine, Primary psychotic disorder, Schizophrenia

## Abstract

Use of amphetamine and methamphetamine is widespread in the general population and common among patients with psychiatric disorders. Amphetamines may induce symptoms of psychosis very similar to those of acute schizophrenia spectrum psychosis. This has been an argument for using amphetamine-induced psychosis as a model for primary psychotic disorders. To distinguish the two types of psychosis on the basis of acute symptoms is difficult. However, acute psychosis induced by amphetamines seems to have a faster recovery and appears to resolve more completely compared to schizophrenic psychosis. The increased vulnerability for acute amphetamine induced psychosis seen among those with schizophrenia, schizotypal personality and, to a certain degree other psychiatric disorders, is also shared by non-psychiatric individuals who previously have experienced amphetamine-induced psychosis. Schizophrenia spectrum disorder and amphetamine-induced psychosis are further linked together by the finding of several susceptibility genes common to both conditions. These genes probably lower the threshold for becoming psychotic and increase the risk for a poorer clinical course of the disease.

The complex relationship between amphetamine use and psychosis has received much attention but is still not adequately explored. Our paper reviews the literature in this field and proposes a stress-vulnerability model for understanding the relationship between amphetamine use and psychosis.

## Background

Amphetamine and methamphetamine (hereafter amphetamines) can prolong wakefulness, increase focus and feelings of energy as well as decrease fatigue. They can produce euphoria, induce anorexia, and be used to treat narcolepsy and attention deficit/hyperactivity disorder (ADHD). Adverse effects include anxiety, aggression, paranoia, hyperactivity, reduced appetite, tachycardia, increased breathing rate, dilated pupils, increased blood pressure, headache, insomnia, palpitations, arrhythmia and others [1].

Amphetamines inhibit dopamine reuptake by interacting with the dopamine transporter (DAT), thereby increasing the concentration of dopamine in the synaptic cleft [2]. Amphetamines also interact with the vesicular monoamine transporter 2 (VMAT2), leading to increasing amounts of dopamine in the cytosol, a possible mechanism of action for the neurotoxicity of amphetamines. Neurotoxic effects are seen also in serotonergic and noradrenergic neurons.

Amphetamines are highly addictive drugs. Both amphetamine and methamphetamine act directly on the mesolimbic dopaminergic “reward system” [3] by inducing release of dopamine, and to some extent norepinephrine, in the synaptic clefts of the Nucleus Accumbens (NAc) and other terminal areas provoking a euphoric state, but also addiction.

Abuse of amphetamines is widespread in the general population [4-9]. It is also common among psychiatric patients [10,11], where a high percentage test positive for amphetamines [12].

There is overwhelming evidence that patients with psychotic disorders have an increased vulnerability to compulsive use of drugs of abuse [13,14], including psycho stimulants like amphetamines [15]. This may be especially true for patients with schizophrenia like disorders [16]. There may be several explanations for this increased co morbidity, but there is convincing evidence from animal studies that this may be due to shared vulnerabilities for both psychosis and drug use disorders [17]. These animal studies also point to possible neural mechanisms explaning the increased co morbidity [18].

The association between amphetamine use and psychosis has received much attention [19], but several questions about this complex relationship remain unanswered. In the following, we review some of these questions and propose a new model for understanding the relationship between amphetamine use and psychosis.

## Prevalence and clinical presentation

Observations strongly suggest a relationship between the intake of amphetamines and the development of acute psychosis. First, early studies demonstrated that amphetamines could trigger acute psychosis in healthy subjects. In these studies, amphetamine was given in consecutively higher doses until psychosis was precipitated, often after 100–300 mg of amphetamine [20-23]. The symptoms subsided within 6 days. The effect was blocked by the use of anti-psychotics [24]. Not all the subjects in these studies became psychotic, as some had to be removed from the experiment because of health risks caused by elevation of heart rate, blood pressure or body temperature. Secondly, psychosis has been viewed as an adverse event, although rare, in children with ADHD who have been treated with amphetamine [25-30]. Thirdly, drug-induced psychosis has been reported in 8–46% of regular users of amphetamines [31-37]. The wide variation is probably due to different populations being studied, gender [38] and the method and duration of amphetamine use [39]. It may also depend on the instruments used to assess psychosis, e.g. self-report [36] vs. formal diagnostic instruments [31,40]. Lastly, there is a positive correlation between amphetamine availability at a community level and the incidence of psychosis in the same population [41-44].

Amphetamine has a terminal elimination half life (T_½_) of 12–15 hours [45] and is often taken several times over the course of many days in runs or binges [46-49]. There is clinical evidence that such binges may end in psychosis. Surprisingly, it is poorly understood [1,50], whether such psychosis is due to amphetamine use *per se* (amount over time, amount on one occasion or the length of the current binge), vulnerabilities in the user or both. It could be that psychosis occurs because of the sleep deprivation that follows amphetamine use, or because of other factors at the end of a binge. Users will often end their binge by using sedating drugs like alcohol, benzodiazepines, opiates or cannabis. This could be viewed as self-medication [51] and may be one reason why users often develop problems with several drugs. Only a weak relationship has been reported between psychotic symptoms and the level of amphetamines in the blood of psychotic patients [51,52]. This could be because acute blood levels at the end of a binge are a poor representation of overall amphetamine exposure, but it could also be because individual vulnerability, rather than level of amphetamine exposure, is the critical risk factor for developing acute psychosis.

The symptoms of psychosis induced by amphetamines are very similar to those of acute schizophrenia spectrum psychosis and include: lack of concentration, delusions of persecution, increased motor activity, disorganization of thoughts, lack of insight, anxiety, suspicion and auditory hallucinations [21,22,53]. Some studies have suggested differences with more pronounced grandiosity and visual hallucinations [52,54]. The thought disorders that occur in schizophrenia characterized by a splitting and loosening of associations, a concreteness of abstract thought, and an impairment in goal-directed thought, may be less prominent in amphetamine induced psychosis [55]. However, distinguishing the two types of psychosis on the basis of acute symptoms is probably very difficult [56]. The similarities between the two conditions are, in fact, so pronounced that this has been used as an argument for using amphetamine-induced psychosis as a model for primary psychotic disorders [21,54,57-59].

In contrast to schizophrenic psychosis, acute psychosis induced by amphetamines seems to have a faster recovery [60-63], and appears to resolve with abstinence, although the recovery may be incomplete [43]. Japanese researchers have argued that psychosis induced by amphetamines could, in fact, be of much longer duration, up to several years [64-66]. This research describes spontaneous psychotic relapses in the long term after remittance of psychosis (“flashbacks”), a phenomenon acknowledged in the popular folk culture in the USA and Europe, but much less researched. Stressful situations seem to trigger such flashbacks in susceptible individuals and several vulnerability factors have been identified, e.g. a family history of psychosis [67-70]. It is difficult to distinguish the Japanese chronic amphetamine psychosis from a primary psychosis triggered by the use of amphetamines [64], although it has been claimed that they constitute separate entities.

### Risk factors and acute vs. chronic psychosis

In animal models, there is sensitization to the rewarding effects of amphetamines (e.g. [71]). Sensitization is also seen in human subjects [72]. There is reason to believe that an earlier psychosis involves a risk of future psychotic episodes due to this sensitization [43,73-75], or possibly to the development of dopaminergic super sensitivity [76,77]. Psychosis may be precipitated acutely by amphetamine due to its effects on dopaminergic activity in the CNS [46]. In the longer term, the neurotoxic effects of the drugs on serotonin and dopamine neurons [78] and dopamine transporters [79] may play a role. Amphetamine sensitization seems to cause dysregulation of dopamine by the ventral subiculum [80]. There is an over-expression of the dopamine receptor, subtype 2 (DRD2) [81] and a higher sensitivity of DRD2 to the effects of amphetamines in vulnerable individuals [82].

In addition to the increased risk of psychosis following the use of amphetamines in people who have experienced amphetamine-induced psychosis previously, patients with schizophrenia [83] and schizotypal personality traits [74,84] may more readily become psychotic after the use of amphetamines. Other risk factors for psychosis may include amphetamine use disorders (abuse and dependence), the presence of other psychiatric disorders (primarily attenuated psychosis syndrome, personality disorders and affective disorders), early cognitive dysfunction (such as those found in the prodromal states of schizophrenia), family history of mental disorder and the use of other drugs like opiates, benzodiazepines, cannabis and alcohol [37,67,74,75,85-87].

Several susceptibility genes have been found in common for amphetamine-induced psychosis and schizophrenia [32,58]. These genes increase the risk both for becoming psychotic and for a poorer clinical course of the disease. Studies in Japan also indicate that primary and drug induced psychosis may be genetically linked. Relatives of methamphetamine-users with a lifetime history of amphetamine psychosis are 5 times more likely to have schizophrenia than methamphetamine-users without a history of psychosis [85]. Patients with schizophrenia and those with psychosis induced by amphetamines both show significantly increased peripheral plasma levels of norepinephrine compared to methamphetamine users who do not have psychosis, and to non-using conrols [65,88]. This seemingly common vulnerability is important considering the difficulties in distinguishing between the two conditions in the long term.

The precipitation of psychosis by amphetamines in healthy subjects can be blocked by anti-psychotics [24,89]. Similarly, psychotic symptoms caused by amphetamines can, like acute schizophrenic psychosis, be treated with anti-psychotics [90]. A Cochrane review from 2009 [91] identified only one randomized controlled trial of treatment for psychosis induced by amphetamines which met the criteria for included studies. It showed that both olanzapine and haloperidol in clinically relevant doses were effective in treating psychotic symptoms [92]. One problem with using anti-psychotics could be that such drugs have a tendency to block the DRD2, potentially increasing anhedonia that could, in turn, cause a greater vulnerability to relapse into drug abuse. Some studies indeed point in this direction [93-95]. The use of alternative therapeutic drugs, such as benzodiazepines, will reduce the chance of extra pyramidal adverse effects [96] and decrease the risk of intoxication [97]. However, one argument against this strategy is that anti-psychotics seem to protect against the neurotoxic effects of amphetamines [98,99].

To further complicate the situation, it seems that up to 25% of those initially diagnosed with drug induced psychosis after some years develope a primary psychotic disorder [100], and even higher figures when looking at methamphetamine induced psychosis [67]. This leaves us with a dilemma - how valid is a diagnostic construct of amphetamine induced psychosis? Such a term suggests the assumption that this type of psychosis can be induced in individuals otherwise not susceptible to, e.g. schizophrenia or other primary psychotic disorders. The ambiguity and difficulties of this diagnosis are reflected in a recent review where the authors suggest the alternative term “substance-associated psychosis” and state that there is a dearth of research that rigorously examines the validity of the diagnostic criteria across substances [101]. These researchers identified 18 papers that specifically focused on delineating the clinical characteristics or outcomes of individuals diagnosed with substance-induced psychosis. Seven of these papers focused on stimulants (amphetamines and cocaine), but only one had a 1 year follow-up assessment.

Also for other substances of abuse with a tendency to cause psychosis there have been similar discussions. It has long been recognized that the use of cannabis in early adolescence increases the risk of later development of psychosis and schizophrenia [102,103]. Because the drug intake takes place many years before the diagnosis of schizophrenia it has been argued that this cannot be a case of reversed causality [104]. There are however some arguments in the opposite direction. Firstly, even with a formidable increase in the use of cannabis in the population, no increase in the incidence of schizophrenia has been observed [105]. Secondly, it has been shown that patients with schizophrenia may have their psychosis triggered by lower intake of cannabis than healthy volunteers [106]. Lastly, we do not know *when* a psychotic disorder starts to develop. It may be that it starts long before the initial psychotic symptoms, opening for the possibility of reversed causality, even when intake of cannabis takes place years before first psychotic episode [104]. The present agreement in the field seems to be that cannabis can precipitate psychosis in vulnerable individuals, an agreement that closely resembles our suggested model. The similarities between amphetamines’ and cannabis’ risk of being associated with later psychosis is further supported by a newly published record linkage study [107].

### A model for the relationship between psychosis induced by amphetamines and primary psychotic disorder

The similarities between acute schizophrenic psychosis and psychosis following the use of amphetamines are so pronounced that the latter has been suggested as a model for schizophrenia [58,108]. However, it remains unresolved whether the relationship between amphetamines and psychosis is explained by drug exposure (amphetamine-induced psychosis), amphetamines use triggering a primary psychotic disorder or both. Although psychosis may be induced by amphetamine in healthy individuals, not all subjects become psychotic by the doses of amphetamines allowed in the experiments. Some, but not all, individuals using amphetamines have experienced psychotic episodes, and a few have experienced psychosis as an adverse event during stimulant treatment. Is this a result of differences in amphetamine exposure or differences in vulnerability? Furthermore, psychosis is precipitated by a lower dose of amphetamines in individuals with primary psychosis and may be blocked by the use of anti-psychotics. Finally, there seem to be many genetic and physiological similarities between amphetamine-induced psychosis and acute schizophrenic psychosis, suggesting that vulnerability may play a significant role in the occurrence of amphetamines-induced psychosis.

**Figure 1:**
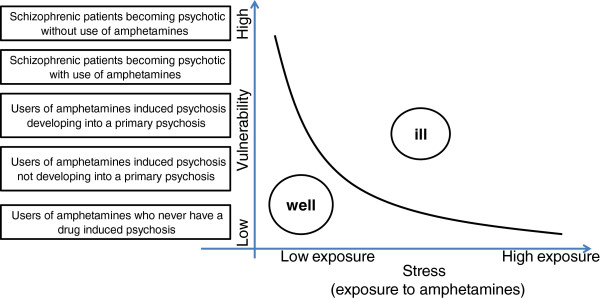
**The relationship between amphetamine use and the development of psychosis can be understood with a traditional stress/vulnerability paradigm.** Some users of amphetamine will not develop psychosis even using high amounts, while others will develop psychosis with little or no exposure.

In this context, we hypothesize that the relationship between amphetamine-induced psychosis and primary psychosis can be viewed within the framework of a traditional vulnerability stress paradigm (Figure 1). Exposure to amphetamines should be viewed as a stressor in the acute phase for the vulnerable individual in a dynamic way; for individuals with lower vulnerability higher doses of amphetamines are needed, whereas individuals with higher vulnerability require lower doses to precipitate acute psychosis. In addition, due to their sensitizing effects, amphetamines may also play a role in the development of vulnerability. Repeated use of amphetamines could increase vulnerability, thereby increasing the chances of developing psychotic symptoms even in the absence of (acute exposure to) amphetamines. Thus, primary psychotic disorder and psychosis precipitated by amphetamines need not be considered as two separate phenomena, but as two phenomena interlinked in a dynamic way.

Taking such a view may have some important clinical implications. Patients diagnosed with drug induced psychosis following intake of amphetamines should be monitored more closely, in particular for signs of a chronic development. Any further use of amphetamines should be strongly discouraged, since it would constitute an acute stressor that could precipitate psychosis and increase the individual’s vulnerability to developing a more chronic psychotic disorder. This model also provides a rationale for future studies of preventive anti-psychotic treatment, including psycho-social, psycho-educational and possibly also pharmacological interventions to decrease vulnerability, in the same way as is recommended for individuals with a primary psychotic disorder.

## Competing interests

None of the authors have any conflicts of interest to declare.

## Authors’ contributions

All the authors have contributed substantially to the idea, structure and content of the paper. All authors have discussed all concepts and formulations in the final manuscript and have approved the submitted versions. JGB has, as first author, written the first draft of all the sections, but these have been altered and reformulated by all the authors in several collaborating rounds.

## Pre-publication history

The pre-publication history for this paper can be accessed here:

http://www.biomedcentral.com/1471-244X/12/221/prepub
